# Factors affecting the persistence of endangered Ganges River dolphins (*Platanista gangetica gangetica*)

**DOI:** 10.1002/ece3.6102

**Published:** 2020-02-13

**Authors:** Shambhu Paudel, John L. Koprowski

**Affiliations:** ^1^ School of Natural Resources and the Environment University of Arizona Tucson AZ USA; ^2^ Institute of Forestry Tribhuvan University Pokhara Nepal

**Keywords:** evolutionary potential, evolutionary traps, freshwater species, Ganges River dolphin, management implications, South Asian waterways

## Abstract

The Ganges–Brahmaputra–Meghna and Karnaphuli (GBMK) River Basin in Nepal, India, and Bangladesh is among the world's most biodiverse river basins. However, human‐induced habitat modification processes threaten the ecological structure of this river basin. Among the GBMK’s diverse flora and fauna of this freshwater ecosystem, the endemic Ganges River dolphin (*Platanista gangetica gangetica*; GRD) is one of the most charismatic species in this freshwater ecosystem. Though a >50% population size reduction has occurred since 1957, researchers and decision‐makers often overlook the persistence (or evolutionary potential) of this species in the highly fragmented GBMK. We define the evolutionary potential as the ability of species/populations to adapt in a changing environment by maintaining their genetic diversity. Here, we review how evolutionary trap mechanisms affect the dynamics and viability of the GRD (hereafter Ganges dolphin) populations after rapid declines in their population size and distribution. We detected six potential trap mechanisms that might affect the Ganges dolphin populations discretely or in combination: (a) habitat modification; (b) occurrence of finite and geographically restricted local populations; (c) ratio of effective to estimate population size; (d) increasing risk of inbreeding depression in genetically isolated groups; (e) at‐risk behavioral attributes; and (f) direct fisheries–dolphin interactions. Because evolutionary traps appear most significant during low water season, they adversely affect demographic parameters, which reduce evolutionary potential. These traps have already caused local extirpation events; therefore, we recommend translocation among populations, including restoring and preserving essential habitats as immediate conservation strategies. Integrative evolutionary potential information based on demographic, genetic, and environmental data is still lacking. Thus, we identify gaps in the knowledge and suggest integrative approaches to understand the future of Ganges dolphins in South Asian waterways.

## Graphical abstract

The Ganges–Brahmaputra–Meghna and Karnaphuli (GBMK) River Basin in Nepal, India, and Bangladesh is among the world's most biodiverse river basin. The endemic Ganges River dolphin (*Platanista gangetica gangetica*; GRD) is one of the most charismatic species in this freshwater ecosystem, which lost 50% of its population size since the last decades. Here, we review how evolutionary trap mechanisms affect the dynamics and viability of this species after rapid declines in their population size. We detected six potential trap mechanisms representing demographics, genetics, environment, and behavioral factors and suggest integrative approaches to understand the future of GRD in South Asian waterways.

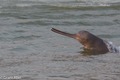

## 1 INTRODUCTION

**Figure 1:**
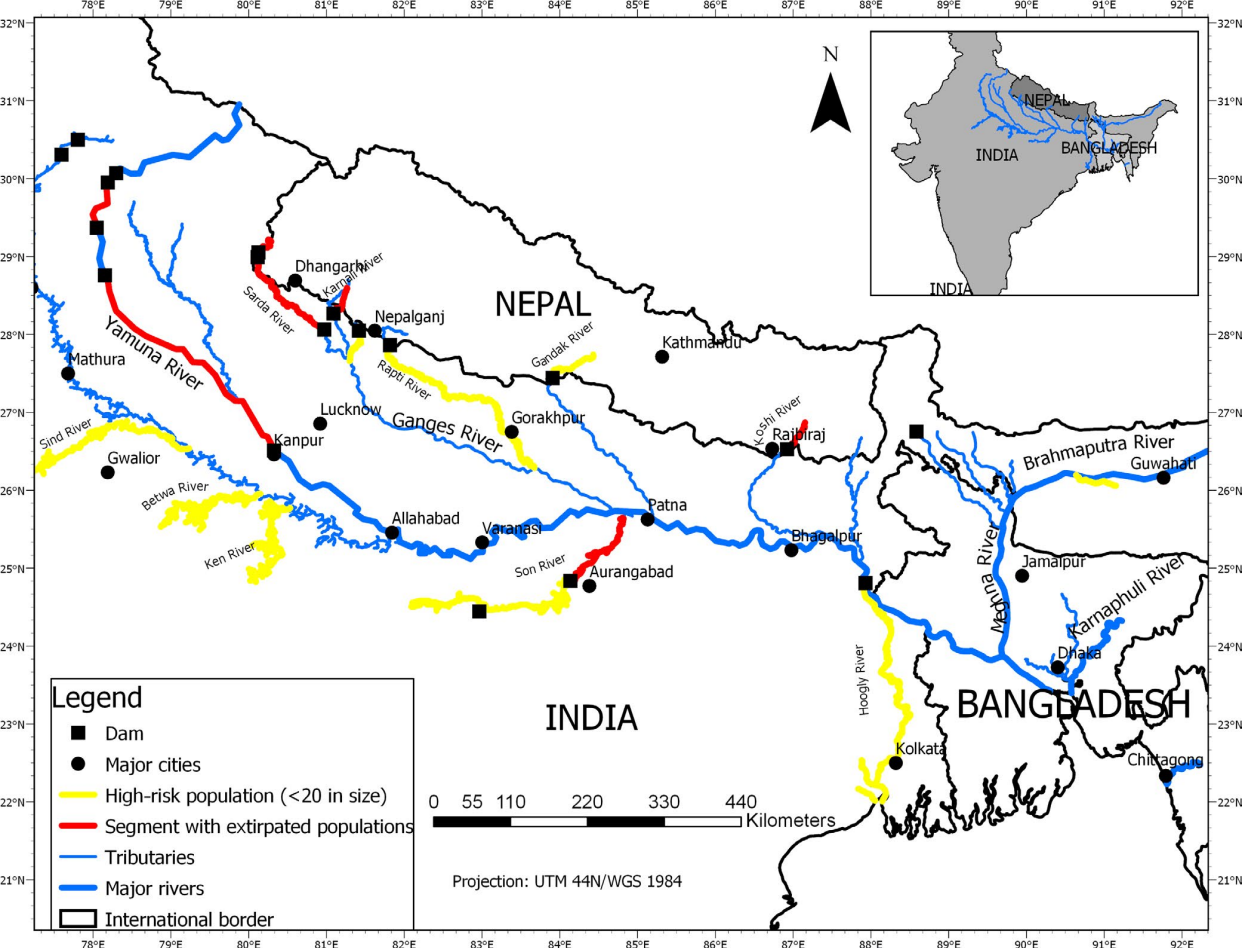
The Ganges–Brahmaputra–Meghna and Karnaphuli Basin in South Asia showing the river dolphin distribution river networks and location of major dams that isolated the dolphin groups. Most of the dams are located at the border between countries resulting in high‐risk small groups. Yellow color indicates the river segment with high‐risk subpopulations (<20 population size), and red depicts segments with the extirpated population

While many landscapes, including freshwater ecosystems, around the world are being transformed by humans at unprecedented rates, understanding the evolutionary potential of endangered species is often ignored (Moritz & Potter, 2013). South Asian Rivers are under threats as almost all the surrounding countries (e.g., Nepal, India, Bangladesh, and China) scramble to harness hydropower and water extraction for expanding agrarian economies (Pereira, Cordery, & Iacovides, 2009). The Ganges–Brahmaputra–Meghna and Karnaphuli River Basin (GBMK; Figure 1) in Nepal, India, and Bangladesh is home to the world's most endangered freshwater river dolphin—the Ganges River dolphin (GRD). Unfortunately, several anthropogenic and natural factors jeopardize the future of this species. The dams and barrages made by the Indian government at or near the international borders (e.g., Nepal–India and India–Bangladesh) threaten the Ganges dolphin ecology by reducing or modifying habitats (Rahman, Rahman, & Asaduzzaman, 2010). These processes present sizeable irreversible extinction risks for Ganges dolphins, similar to those that contributed to the recent human‐induced extinction of the Yangtze River dolphin (Turvey et al., 2007). However, the extinction risk for the Ganges dolphin is poorly understood, and how small isolated groups of the Ganges dolphin will respond to novel environmental changes while maintaining genetic diversity is unknown. While habitat loss and fragmentation are the most critical documented factors (Paudel, Timilsina, Lewis, Ingersoll, & Jnawali, 2015; Sinha & Kannan, 2014), a combination of several factors likely put the Ganges dolphin at risk of extinction. Different factors may act, and interact, to drive the Ganges dolphin populations to extinction by reducing population sizes and stability and creating downward cycles to extinction demographically. Our ability to understand the extent and potential magnitude of such threats is limited, yet essential to develop an integrative conservation strategy in a changing environmental setting.

A large number of dams and water‐related projects are planned or under construction in the main reaches and tributaries of the GBMK (Verma, Kampman, Zaag, & Hoekstra, 2009). Such flow‐regulating barriers (e.g., 19 hydropower dams and 23 barrages; Figure 1) were created from the 1950s through the 1980s in the GBMK (Smith et al., 2000). Barriers not only contract the range of the Ganges dolphin distribution but also create small, local subpopulations, which reduces the evolutionary potential of the Ganges dolphin. Such small subpopulations with restricted gene flow are exposed to increased inbreeding and loss of genetic diversity, likely leading to higher extinction risks. Genetically isolated small subpopulations of the Ganges dolphin have already been extirpated from some river segments in the GBMK Basin (e.g., upstream of Gandak and Sapta Koshi Rivers in Nepal). In such an intensively human‐modified landscape, several evolutionary traps (i.e., an adaptive trait suddenly becomes maladaptive, leading to extinction) are more prominent and hinder the further process of the Ganges dolphin evolution (Robertson, Rehage, & Sih, 2013; Schlaepfer, Runge, & Sherman, 2002).

Though the Ganges dolphin is one of the most endangered cetaceans and constantly under pressure throughout the GBMK Basin, the evolutionary potential of the Ganges dolphin has not yet been assessed. As the species faces severe threats of extinction, understanding evolutionary potential helps us to predict the rate of adaptation to ongoing environmental change. Our review will evaluate the genetic stability of the Ganges dolphin and its adaptation to the changing environment. Because of several threatening ecological and physiological traits (e.g., highly seasonal migration in relation to flow, Anderson, 1879; patchy distributions with finite habitat preference, Bashir, Khan, Gautam, & Behera, 2010; Paudel, Pal, et al., 2015; small litter sizes, Anderson, 1879; and solitary behavior; see details in Sinha & Kannan, 2014 for biology, ecology, and conservation status of the Ganges dolphin), the comprehensive evaluation of the Ganges dolphin evolutionary potential could improve our understanding of their potential viable populations.

The few previous conservation studies of the Ganges dolphin examined habitat components (Khanal et al., 2016; Smith & Reeves, 2012), feeding and foraging behaviors (Kelkar et al., 2018), and flow regimes (Choudhary et al., 2012; Khanal et al., 2016). Critically, these previous efforts did not integrate ecological, demographic, and environmental factors that might accelerate extinction risk before genetic deterioration (Lande, 1988). In response to this issue, ecological and evolutionary information is critical while predicting species extinction risks. A fifty per cent reduction in global population size since the 1990s along with increasingly isolated populations exacerbates extinction risks for the endangered Ganges dolphin (Braulik & Smith, 2017). Thus, an accurate account of how the Ganges dolphin responds to a changing environment is fundamental to the establishment of meaningful conservation actions, including managing reintroductions, and long‐term monitoring.

In this review, we collected and analyzed demographic, behavioral, environmental, and genetic‐based information in an integrative way to predict the evolutionary potential of the Ganges dolphin in South Asian waterways. We highlight possible mechanisms that could affect the dynamics and viability of the Ganges dolphin populations throughout their range in Nepal, India, and Bangladesh. To our knowledge, this is the first study that demonstrates the effects of genetic, demographic, and environmental factors on the evolutionary potential of small isolated subpopulations of the Ganges dolphin. This information could be applied to forecast the fate of endangered small cetaceans (i.e., river dolphins in south/east Asia and South America) along with developing integrative conservation management strategies for waterways they inhabit. We also identify research gaps that hinder our understanding of the Ganges dolphin evolution and ecology.

## 2 MATERIALS AND METHODS

We reviewed published peer‐reviewed journal articles and books related to the Ganges dolphin from January 1900 through December 2018 through the “Google Scholar” and Thomson Reuters “Web of Science” databases. We refined our review by using the following keywords in different combinations: GRD, evolution potential, demographic, environmental process, behavior, dams/barrage, catastrophic, and genetic viability. We found 85 publications comprised of journal articles (*n* = 61), review articles (*n* = 4), notes (*n* = 2), proceedings articles (*n* = 16), and book chapters (*n* = 2) related to the Ganges dolphin population (*n* = 20), biology (*n* = 12), ecology (*n* = 7), anthropogenic threats (*n* = 12), echolocation and communication (*n* = 14), phylogenetics (*n* = 6), evolution (*n* = 2), biochemistry (*n* = 8), anatomy (*n* = 3) and disease (*n* = 1). We did not find publications devoted to interactive evolutionary potential of the Ganges dolphin; however, two partially dealt with the integrative evolutionary potential of the Ganges dolphin (Kelkar et al., 2018; Smith & Reeves, 2012).

We grouped all the potential persistence‐threatening processes under six potential evolutionary trap mechanisms that might drive the evolutionary potential of the Ganges dolphin in the GBMK Basin: (a) habitat modification (change in the Ganges dolphin preferred hydrophysical habitat in terms of feature [e.g., pool, riffle, and run] and quality [e.g., depth and velocity]); (b) occurrence of finite and geographically restricted local populations; (c) ratio of effective to estimate population size (*N*_e_ < 500); (d) increasing risk of inbreeding depression in genetically isolated groups; (e) at‐risk behavioral traits; and (f) direct fisheries–dolphin interactions (Table 1). We describe and discuss each mechanism individually; however, we merged genetic mechanisms (i.e., traps #3 and #4) to present potential relationships between genetic isolation and risk of inbreeding in the Ganges dolphin. Because of a lack of knowledge on potential evolutionary trap mechanisms of the Ganges dolphin, we focused on identifying evolutionary trap mechanisms that represent demographic, genetic, and environmental factors and suggest future study to prioritize these mechanisms and their interactive effects. We used the ratio of effective (*N*_e_) to estimate population size (*N*_c_) to quantify the relative rate at which genetic diversity eroded, a fundamental process of evolutionary change (Frankham, 2003). Knowledge of the relative magnitudes of these two parameters is essential to understand population persistence, mainly because *N*_e_ is generally much lower than *N*_c_ in natural populations (Frankham, 1995). Here, we estimated long‐term historical *N*_e_ as an informative tool to determine the risk of population extinction assuming average ratio value (*N*_e_/*N*_c_) = 0.11 (Frankham, 1995). To reduce the potential risk of redundancy and to make findings more interactive, we discuss our results under four headings that cover identified evolutionary traps mechanisms: physical habitat modification, genetics, Ganges dolphin behavioral ecology, and dolphin‐fisheries interactions. Maps related to distribution, isolated, and extirpation segments were prepared using ArcGIS Pro 2.3.2 (Environmental Systems Research Institute [ESRI Inc.], 2018). We also used the Ganges dolphin natural picture from the field to support a hypothesis that we deduced from literature reviews.

**Table 1:** The different mechanisms and their associated processes, and types of mechanism that might affect the dynamics and viability of the Ganges River dolphin (Ganges dolphin)

Mechanisms thought to drive evolutionary potential	Type	Processes that might affect the dynamics and viability of the Ganges dolphin	Persistence scale of species
Habitat (or quality) modification	E/A	Because of high tropic level, dolphins migrate to their preferred environmental optima or adapt in situ to avoid extinction. Resulting extirpation or local small groups of population	Low
Occurrence of finite and geographically restricted local populations	A	Increase homozygosity and the expression of deleterious recessive alleles; loss of allelic diversity at functional genes; and thus increase risk of inbreeding	Low
Ratio of effective to estimate population size (<500)	D/G	Increase risks of inbreeding and genetic drift affecting the adaptive potential of species	Low
Risk of inbreeding in small local populations	G	50% reduction of population size over three generations; loss of genetic diversity in small populations reduce the ability of population to evolve with environmental change, as genetic diversity acts as raw materials for adaptive evolutionary	Low
At‐risk behavioral attributes (including biological attributes)	B/I	Adaptive behaviors can become maladaptive in the new setting and eventually caught in an evolutionary trap. Rates at which behaviors realign themselves after being caught in a trap depend on the strength of selection imposed by the trap and the degree to which the behaviors are phenotypically plastic. Looking at localized habitat preference, taking water level as cues, nongregarious trait, fish removal from gillnet could reduce potential persistence scale.	Variable
Increased fisheries–dolphin interaction	A	Increase mortality rate reduce the potential of evolution by increasing the cost of survivorship	Variable

Note

Based on the animal sensitivity to the mechanism, scaled persistence was assigned to each mechanism involved (type of mechanism: E—environmental; A—anthropogenic; D—demographic; G—genetic; B—behavioral; I—intrinsic).

## 3 RESULTS

We found only two papers that partially incorporate the evolutionary potential of the Ganges dolphin in response to a changing environment. Kelkar et al. (2018) focused on adaptive capacity by combining anatomy, physiology, and morphology. Further, Smith and Reeves (2012) assessed whether the Ganges dolphin is a specialist or generalist using systematic animal taxonomy and behavioral facts to understand their adaption in a human‐modified landscape. Our review of potential mechanisms that might hinder the evolutionary potential of the Ganges dolphin is defined in detail below.

### 3.1 Habitat modification

When habitat was modified, we noticed local extirpation and spatial disequilibrium of isolated subpopulations of the Ganges dolphin increased (Paudel, Pal, et al., 2015; Sinha & Kannan, 2014). Aquatic animals have adapted to existing cycles of silt, nutrients, and discharge. Land use strongly influences the habitat quality of streams and rivers (Allan, 2004). Therefore, a dramatic transformation of landforms into human uses, together with tripling human population size, in South Asia might alter the hydrophysical habitats in South Asian waterways (Richards & Flint, 1994). With such massive land transformation and increased social pressure, presumably characteristics of mesohabitats available in the GBMK Basin change through alterations of the magnitude of discharge, pollution, sedimentation, and riparian attributes. As a result, foraging grounds and migratory pathways might block, fragment, or destroy the Ganges dolphin populations (Dudgeon, 2000). This further exacerbates ongoing Ganges dolphin isolation and habitat fragmentation in the GBMK Basin. We identified six extirpated populations in various rivers segments (Table 2)—an 18% reduction in the global distribution range of the Ganges dolphin—and nine geographically isolated small groups (with the possibility of connectivity) that might have resulted from this trapping mechanism (Table 3).

**Table 2:** Details of complete populations’ extirpated segments in the GBMK River Basin with their length size

Country	Location of population's extirpated segment	Length (km)
India	Between Haridwar and middle Ganga Barrage	100
	Lower Ganga Barrage to Kanpur	358
	Sone River to its confluence with Ganga	300
	Sharda River	100
Nepal	Upstream Mahakali River from Sharda Barrage	40
	Upstream from Sapta Koshi Barrage	49

Abbreviation: GBMK, Ganges–Brahmaputra–Meghna and Karnaphuli.

**Table 3:** Questionably viable groups in the GBMK River Basin that require immediate conservation action with their population size

Country	Location/river segment	Length (km)	Estimated population size in a river segment
India	Rapti River	20	8
	Surya River	22	16
	Between Bicchi in Madhya Pradesh to Banjari	130	10
	Ken River (to Yamuna and Sindhan confluence)	30	8
	Betwa (confluence with Yamuna to Orai)	84	6
	Sind (confluence with Yamuna to 110 km upstream)	110	5
	Rupnarayan (Gadiara to Mankar)	424	18
	Kulsi (from Gharamara to its confluence with the Brahmaputra at Nagarbera)	76	17
Nepal	Narayani (above Gandak Barrage)	85	2

Note

These groups were isolated either by the effect of dams (water extraction effect in the downstream) or naturally reduced habitat (low water level, mainly in the upstream tributaries of the Ganges River) and are reported historically from the region as localized population.

Abbreviation: GBMK, Ganges–Brahmaputra–Meghna and Karnaphuli.

**Figure 2:**
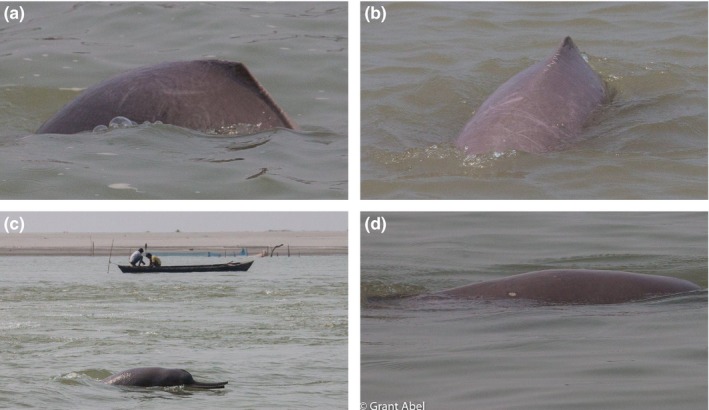
Skin lesions (a, b, d) and physical injured (c) recorded on the Ganges dolphin below Sapta Koshi Barrage in Nepal. Highly fluctuating hydrophysical properties and acute interaction with fishing nets might be the reasons that increase dolphins’ susceptibility to skin‐related disease

Water pollution due to land transformation might also adversely affect the health of the Ganges dolphin populations. The Ganges dolphin's cannot adequately metabolize contaminants (Senthilkumar, Kannan, Sinha, Tanabe, & Giesy, 1999) and might suffer from skin, reproductive, and immunological diseases from water pollution (Acevedo‐Whitehouse & Duffus, 2009; Colborn & Smolen, 1996; Helle, 1976; Kannan et al., 1993; Van Bressem et al., 2009). We observed several instances of skin lesions (e.g., a circular scar on the back, long scars on the abdomen, cross‐hatch like scars on the dorsal ridge and dorsal fins) in most animals sighted below the Sapta Koshi Barrage in Nepal (Figure 2; personal observation). Furthermore, Senthilkumar et al. (1999) linked a reduction in abundance of the Ganges dolphin in the Ganges River to a higher level of contamination (e.g., DDTs [dichlorodiphenyltrichloroethane] and PCBs [polychlorinated biphenyls]). Similarly, Behera, Singh, and Sagar (2013) also found pollution as a cause of local extirpation in the middle Ganges River (between Kachlaghat and Kanpur) of India.

### 3.2 Occurrence of finite and geographically restricted local populations

**Figure 3:**
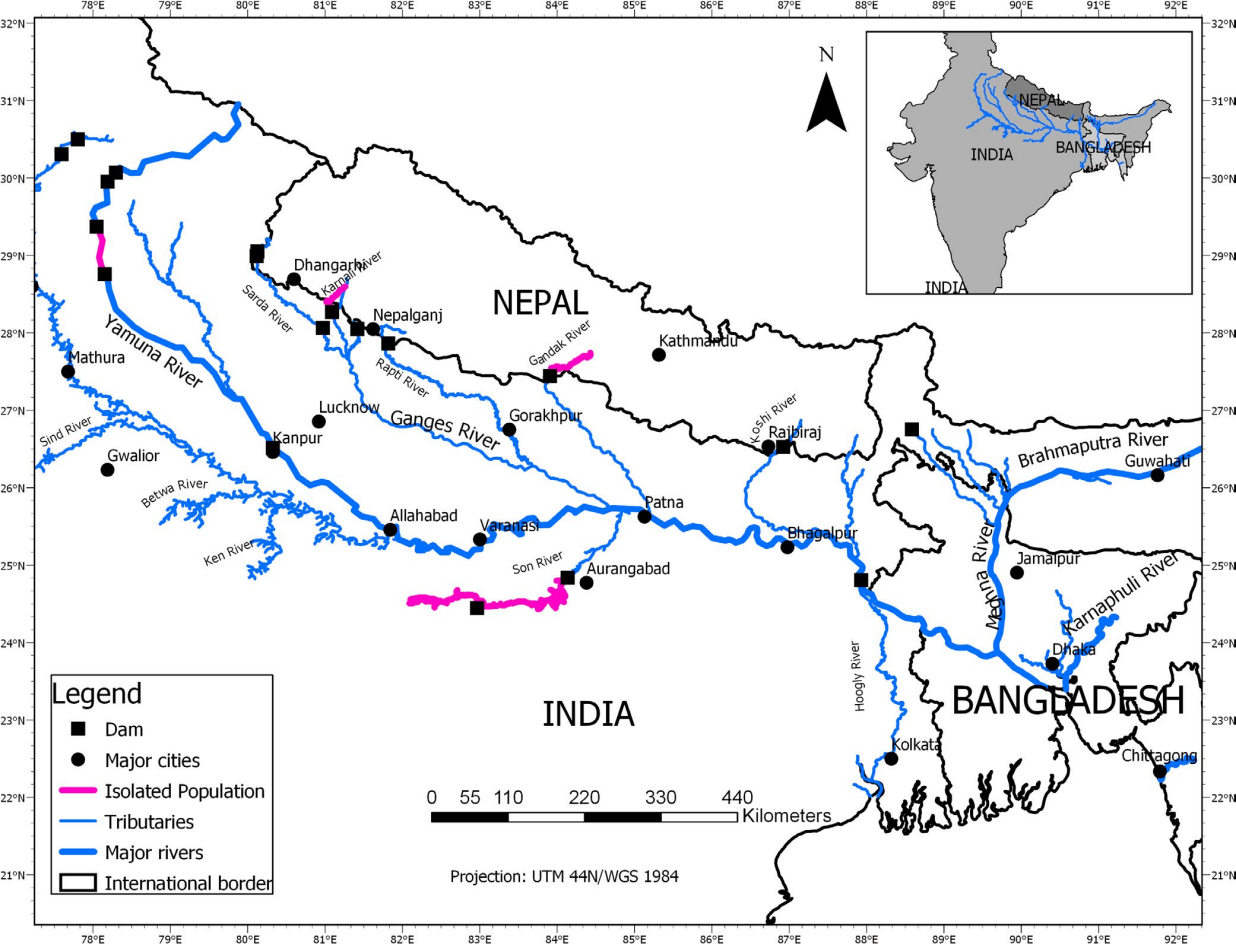
Genetically isolated groups (which might overlap with high‐risk groups) of Ganges dolphins by dams/barrages in Nepal and India rivers’ segments. As a result, dolphins in the upstream tributaries are more sensitive, and as result, most extirpated segments were reported only from the upstream of the Ganges

We identified various water development projects in the GBMK Basin (Nepal = 8; India = 42; Bangladesh = 16; Figures 1 and 3) that affect rivers historically or currently supporting the Ganges dolphins. These large structures across rivers have contributed to local extinction and genetically isolated small local populations of the Ganges dolphins with small geographic ranges in the GBMK Basin. We found four genetically restricted subgroups in Nepal and India with small effective geographic ranges, and possibly unidirectional movements (Figure 3; Table 4). Higher effects of fragmentation occur in the major tributaries (e.g., Nepal and India) of the Ganges River compared to rivers in Bangladesh (Sinha & Kannan, 2014; Smith et al., 2000). For example, the Girijapuri Dam located 20 km below the Nepal/India border in the Karnali River of Nepal offers <30 km of effective upstream habitat, with a subpopulation size smaller than 50 individuals. Out of 5,317 total km of available stream habitat in the GBMK, the Ganges dolphin was completely extirpated from 18%. Further, we found nine naturally isolated subpopulations (possibility of interconnectivity depends on water level), with group sizes smaller than 20 individuals (Table 3; Figure 1; Sinha & Kannan, 2014). These subgroups localized in a limited area of the river segment because of the nonlinear hydrophysical habitat.

**Table 4:** Locations of genetically isolated groups of the Ganges dolphins in the GBMK River Basin with their group size

Country	Location of isolated group	Estimated group size
India	Between middle and lower Ganga Barrage	56
	Between Bicchi in Madhya Pradesh to Banjari in Bihar	10
Nepal	Above Gandak Barrage	2
	Above Girijapuri Barrage in India to upstream Nepal	50–60

Note

The dams/barrages isolate these groups.

Abbreviation: GBMK, Ganges–Brahmaputra–Meghna and Karnaphuli.

### 3.3 The ratio of effective to estimate population size and increasing risk of inbreeding depression

Roxburgh (1801) made the first documentation about the Ganges dolphin. Then, Anderson (1879) reported on the distributional range, morphology, and anatomy of this species. Approximately 100 years later, only a few papers offered further details on the population status of the Ganges dolphin (Jones, 1978; Lal Mohan, 1989). Its global population size was estimated to be 4,000–5,000 in the GBMK Basin (Jones, 1978). The species had declined to 3,526 in 2014 (Sinha & Kannan, 2014), indicating a 30% loss in four generations (assuming a 9‐year generation rate). We estimated *N*_e_ of 550 with an estimate size of 5,000 for the year of 1982 (Jones, 1978) and 388 for 2014 (Sinha & Kannan, 2014). This value reveals that the Ganges dolphin is suffering from insufficient effective population sizes (*N*_e_ < 500) required to maintain viable genetic diversity (Franklin, 1980). Because of such a small current *N*_e_ (≤500) and fragmented into isolated small subpopulations, inbreeding depression could be the most immediate and significant hindrance to the evolutionary potential of the Ganges dolphin (Vilas, San Miguel, Amaro, & Garcia, 2006).

### 3.4 At‐risk behavioral traits

The GBMK Basin undergoes seasonal flow patterns that define and inhibit access of the Ganges dolphins to particular foraging or surfacing grounds. The GBMK’s natural flow regime provides Ganges dolphins cues to migrate, reproduce, forage, etc.; thus, the rhythm of the river is tied intimately to the functional ecology of this animal. In the context of rapidly changing environmental settings, such Ganges dolphin dependence on water level could be maladaptive. An inability to adapt to these changes not only subjects Ganges dolphin to an evolutionary trap but also reduces their evolutionary potential by affecting their important life‐history stages (e.g., resting period, preparation for reproduction period). At‐risk behavioral activities (e.g., habitat specialization) in combination with different biological traits, such as small litter size, late sexual maturity, and long gestation period, might also reduce persistence of the Ganges dolphin (Purvis, Gittleman, Cowlishaw, & Mace, 2000). The surfacing and foraging behavior of Ganges dolphins are mostly confined to deep pools and eddies, which provide critical shelter and large prey species (Paudel, Pal, et al., 2015; Paudel, Timilsina, et al., 2015). Because of such habitat sensitivity (e.g., selection of vertical water column and water velocity; Dudgeon, 2000), the Ganges dolphins may hinder their evolutionary potential by reduction of genetic diversity.

### 3.5 Fisheries–dolphin interactions

Increasing pressure from artisanal fisheries heightens the potential for river dolphin and fisheries interactions, mainly through direct competition for certain fish size‐classes as well as habitat and diel activity overlap. Conflicts between Ganges dolphins and artisanal fishing increased dramatically in recent years across the GBMK Basin (Kelkar et al., 2018; Kelkar, Krishnaswamy, Choudhary, & Sutaria, 2010; Paudel, Levesque, Saavedra, Pita, & Pal, 2016). Although by‐catch data were not available for the Ganges dolphin, this is considered one of the prime consequences of the direct fisheries–dolphin interaction (Read, 2008), which further threatens their survivorship of the Ganges dolphins, especially young calves. For example, two young calves (<20 kg) entangled in a gillnet in the Sapta Koshi River of Nepal between 2013 and 2015 (Paudel, 2017).

## 4 DISCUSSION

### 4.1 Physical habitat modification

Altering natural streamflow imperils native biodiversity and increases freshwater functional complexity (Dudgeon, 2000). Given that Ganges dolphins show a strong preference for particular habitats, the presence of small finite Ganges dolphin groups with limited geographic range indicates that they are under severe risk of habitat loss and fragmentation (Fahrig, 1997). Although there is a lack of research quantifying and characterizing the Ganges dolphin breeding habitats, studies of closely related species suggest that loss of suitable hydrophysical habitats (e.g., required depth) might affect the Ganges dolphins indirectly by reducing or eliminating their reproductive success (Robinson et al., 1992). For example, the presence of suitable habitats (e.g., water depth) significantly predicts female reproductive success in bottlenose dolphins (Mann, Connor, Barre, & Heithaus, 2000). To improve survival prospects of the Ganges dolphin, we must, therefore, identify suitable hydrophysical habitats that contribute to its demographic processes and increase efforts to reduce habitat loss. Since the 1990s, researchers have recommended conservation efforts that focus on recovering declining populations in their natural habitats (e.g., Smith, 1993), yet efforts to date have failed to make an impact. Instead, local extinction rates and isolated small population groups have increased (Tables 3 and 4), mostly in the upstream range of the Ganges River. Among all the groups, the Nepalese subpopulations size is too small to support long‐term viability (Paudel, Timilsina, et al., 2015). Because of the heightened risks in such small isolated groups, these subgroups need an immediate effective conservation plan. The need of effective conservation plans for such small isolated populations is clearly illustrated by the recent conservation status of small cetaceans like the Hector's dolphin (*Cephalorhynchus hectori*) in New Zealand, the Indo‐Pacific humpback dolphin (*Sousa chinensis*) in Taiwan and Hong Kong, and the Irrawaddy dolphin (*Orcaella brevirostris*) in Asia (Cagnazzi, Parra, Westley, & Harrison, 2013). Even though the Indian government declared the Ganges dolphin as the national aquatic animal (Sinha & Kannan, 2014) and formulated the Conservation Action Plan for the Ganges dolphin (2010–2020), structures made by the Indian government at international borders have highly modified the environmental structure of the GBMK, thus threatening the same species they declared important. Despite several initiated river dolphin‐based conservation projects (e.g., Vikramshila Gangetic Dolphin Sanctuary, Bhagalpur District of Bihar, India; WCS dolphin conservation project in Bangladesh), our findings suggest more science‐driven regional level conservation initiatives are needed.

Although the loss of the Ganges dolphin is largely attributed to dams/water‐based development structures, we clearly noticed evidence of population increase in some segments, such as between Bijnor (middle Ganges Dam) and Narora (lower Ganges Dam) (Sinha & Kannan, 2014). This implies that effects of fragmentation by dam/barrage are trivial in comparison with the effects of habitat loss (Fahrig, 1997). However, wildlife managers and policymakers often overlook the issue of the quality and quantity of the foraging habitats that influence the Ganges dolphin's reproductive success between barriers. While the reproduction only occurs in suitable foraging grounds, it is also imperative to determine the minimum amount of habitat that needs to be preserved to allow for the persistence of the Ganges dolphin. The combination of needs for both reproduction and persistence means that maintenance of habitat quality is critical for the long‐term survival of the Ganges dolphin. Based on the increased Ganges dolphin population size between dams, further study is warranted because the effects of large water‐based structures, like dams, could be minimal if we sustain the required water levels that allow animal movement and reproduction between barriers. This review stressed that barriers (e.g., dams/barrage) are not a single cause but rather an interaction of multiple issues (e.g., flow release plan, fisheries–dolphin interactions) causing the Ganges dolphin declines. Additional systematic studies that integrate habitat quality assessment, population census, and a genetic approach could support our findings.

### 4.2 Genetic

The most notable barrage is the Farakka Barrage, which divides the global population of the Ganges dolphins at approximately the center of their geographic range, making several regional subpopulations. The Girija, Gandak, upper Sharda, and Koshi Barrage further isolate dolphin populations in their furthest upstream range in Nepal, in which some groups already became extirpated. Because of weak selective pressure in small and isolated populations, animals might tolerate inbreeding despite the cost (Rioux‐Paquette, Festa‐Bianchet, & Coltman, 2010). Since the aquatic mammals are sensitive to the effects of dams (Wu et al., 2004), the questionably viable subpopulations in a limited geographic range (Tables 2 and 4; Figures 1 and 3) are more prone to immediate extinction. This occurs in different ways that ruin genetic diversity (e.g., inbreeding depression, and reduction of genetic variability) and reduce the fitness of the population overall. The addition of each new dam further fuels the fragmentation of habitat and development of small local subpopulations. In such scenarios, where genetic heterozygosity declines and inbreeding increases, the risk of the Ganges dolphin local extinctions seems inevitable. Since species extinctions proceed more rapidly in freshwater than terrestrial environments (Ricciardi & Rasmussen, 1999), we expect to find many small local subpopulations and continued extirpation in the future. However, the rates of decreasing genetic diversity and increasing inbreeding are still unknown and should be further studied.

As demographic and environmental stochasticity drive small populations to extinction before genetic erosion (Lande, 1988), recent local extinction of subpopulations from certain regions, and an increase in questionably viable subpopulations might be attributed to demographics (e.g., fluctuation in population size) and environmental factors. Presently, the Ganges dolphins might not face immediate extinction but are under imminent risks. They might suffer from the gradual depletion of genetic diversity, inbreeding within local groups, and reduced fitness. The declining pattern of *N*_e_ eventually reduces the ability of the Ganges dolphin to adapt to novel environmental threats. This pattern can be used to predict the rate of adaptation of the Ganges dolphin in the South Asian rivers, but we acknowledge this as uncharted territory in the prospects of evolutionary potential. It is true that relying exclusively on estimate data for estimating effective population size might underestimate *N*_e_ value. However, estimate data as a proxy to determine cues of genetic loss or extinction risk could be a reasonable option to interpret population health in data crises in regions like the GBMK Basin. Curtailing the decline of *N*_e_ is critical for the conservation of this endangered species, and thus, it could be useful to urge concerned authorities in South Asia. In particular, the most striking finding of this analysis is the declining and inadequate *N*_e_ of the Ganges dolphin populations. Effective population size can be depressed due to fluctuation in population size, including a variety of biological traits, such as high endemism, long gestation period, small litter size, unequal sex ratio, and variance in group size (Frankham, 2003; Jones, 1978). Further, *N*_e_ is likely to reduce decrease in such small local subpopulations with short geographic range by limited access to suitable foraging sites that may influence the reproductive success of the Ganges dolphin. As a consequence, the number of individuals contributing to reproduction may be less than predicted.

A vital population management implication of low and declining *N*_e_ populations is that substantial population sizes are required for long‐term maintenance of genetic variation (Frankham, 1995). Conservation actions may take the form of more benign environments or managing dolphins to increase reproduction and survival. If heterozygosity loss is capped at 5% or less in the next 50 years, hope to sustain the Ganges dolphin populations still exists. The relatively small and isolated Ganges dolphin subpopulations may require individual translocation among subpopulations to maintain genetic variation. Also, human interventions (e.g., habitat management or preservation activities) to ensure their survival and reproduction with sufficient water level at the proper time (e.g., December–April, peak breeding period) are urgently required. Jones (1975) suggested a similar management approach, the translocation of animals to the Chambal and Rihand tributaries of the Ganges River in India. Smith (1993) also stressed habitat intervention along the stretch of Karnali River (below the Chisapani Bridge) to manage the remaining questionably viable population to prevent its extinction.

### 4.3 Ganges dolphin behavioral ecology

Long‐term persistence of specialist species has been adversely affected by current global and local environmental changes (Clavel, Julliard, & Devictor, 2011). During the past decades, several studies revealed declines in specialist mammals (e.g., Fisher, Blomberg, & Owens, 2003). Given that the Ganges dolphin is more specialized in its circadian rhythms concerning habitat selection (e.g., depth profile selection for reproduction and foraging), human impacts to its habitat may are likely to affect these use patterns that might in turn affect their functional ecology and important life‐history stages. Hydrological cues highly guide the Ganges dolphin functional activities, like locating prey species and reproductive success, which particularly affect functional ecology of the Ganges dolphin (Smith & Reeves, 2012). Further, the Ganges dolphin adopts seasonal movement patterns between the mainstream and tributaries using a cyclic range of water levels. For instance, presence of high‐water flow in the mainstream reach stimulates the Ganges dolphins to migrate to other tributaries (Paudel, Pal, et al., 2015). In certain instances, water regulation by anthropogenic structures (e.g., hydropower dams or development structures) could falsely present as an environmental cue, rendering these evolutionary responses maladaptive. Such evolutionary traps could be more effectively studied in certain locations (e.g., seasonal migration patterns to the Mohana tributary of the Karnali River of Nepal, and habitat occupancy below the Sapta Koshi Barrage). Further, Braulik et al. (2015) found low mtDNA variability in the Ganges dolphins, indicating habitat‐specific behavior or more localized occupancy behaviors might further contribute to the loss of genetic diversity. However, they argued that this could be the result of interactive effects of low population sizes and localized sensitive behaviors. Using different behavioral strategies adopted by the Ganges dolphin, in combination with extinction‐promoting traits, presumably, render Ganges dolphins species extremely vulnerable to extinction.

### 4.4 Human–dolphin conflicts

Interactions between artisanal fisheries and the Ganges dolphin is one of the most significant conservation concerns in the GBMK Basin, leading to endangerment and extinction (Read, 2008). The recent extinction of the baiji (*Lipotes vexillifer*), a freshwater dolphin endemic to the Yangtze River, China, showcases how such interactions can cause dramatic declines (Turvey et al., 2007). Unsustainable by‐catch in local fisheries attributed to the loss of baiji. Similarly, the vaquita (*Phocoena sinus*) with <30 individuals, and 95 Indus River dolphins (*Platanista gangetica minor*) in Pakistan killed in a fishing gear between 1993 and 2012 were all associated with human–dolphin interactions (Jefferson, 2019). Dietary and diel activity, and spatial and temporal overlap with fisheries could be the reasons for Ganges dolphin endangerment associated with fisheries (Read, 2008). Thus, interactions between the Ganges dolphin and subsistence fisheries are a serious and growing problem, and effective management requires an assessment of the factors driving these interactions. Quantitative and qualitative documentation of drivers (e.g., dietary competition, spatial overlap, behavioral distractions, etc.) that lead to negative fisheries–river dolphin interactions could help better manage and promote coexistence between fisheries and river dolphins.

### 4.5 Future management implications

Because of conservative policies and limitation of resources, there currently exists a void in the application of genetic tools to explore the population viability of the Ganges dolphin in the GBMK Basin. Initiating a regional intergovernmental project promoting genetic‐based research to examine genetic viability and factors associated with the risk of extinction is essential. As the Ganges dolphin is facing serious population loss issues, use of noninvasive tools, like environmental DNA, for genetic monitoring might be effective (Foote et al., 2012). But understanding the viability of such a tool is imperative before its application. Conservation projects that build transboundary cooperative mechanisms (e.g., joint venture conservation initiatives among countries) on upstream tributaries of the Ganges and Brahmaputra rivers could promote restoration of the Ganges dolphin hydrophysical habitat. Nepalese and Indian (Uttar Pradesh) authorities should not wait for critical situations, learning from the extinction lessons of the Indus River dolphin (*Platanista gangetica minor*) and Yangtze River dolphin (*Lipotes vexillifer*). Since we noticed a large fluctuation in population size, we emphasize the importance of understanding this fluctuation effects on demographic structure, which might produce a minimal value of the ratio of effective to census population size. Because of weak selective pressure in a small and isolated population, Ganges dolphin subpopulations might be severely affected by the risk of inbreeding and thus could exhibit a unique genetic structure. Using modern gene sequencing methods would improve our ability to test articulated assumptions, we made in this article (e.g., Parsons, Noble, Reid, & Thompson, 2002). We suggest the integration of genetic data with census data to predict more accurate population trends of the Ganges dolphin. If we have a better population genetic knowledge, we could develop appropriate “genetic rescue” to recover this declining species into its natural habitat. At this point, one potential “genetic rescue” tool to improve genetic stability could be translocation of individuals among subpopulations by developing proper capture and handling techniques (Krützen et al., 2018).

From a conservation perspective, our findings imply that making extinction predictions from a single ecological factor may be risky because of synergistic effects of several factors. Thus, we highlight the integration of genetics, demographics, and environmental factors in future studies, which can aid future research and provide a better understanding of conservation and management purposes. In general, isolated, small subpopulations warrant immediate conservation attention, regionally and internationally. Maintenance of minimum stream flow and restoration and preservation of essential foraging and surfacing habitats appear to be the best methods to limit or prevent any further declines of the Ganges dolphin.

## 5 CONCLUSIONS

The evolutionary potential of the Ganges dolphin in the GBMK Basin may be hindered by several mechanisms like spatial and genetic isolation, small *N*_e_ group size, risky behavioral activities, direct dolphin‐fisheries interaction, and habitat modification. As an interactive function of these different mechanisms, we note the reduced evolutionary potential of the Ganges dolphin in the fragmented South Asian waterways as Ganges dolphins are vulnerable to changing environments. Despite this, recent research mostly focuses on discrete conservation issues, and science‐based integrative knowledge of the Ganges dolphin evolutionary potential remains limited. Populations with such spatial disequilibrium can have significant ecological and evolutionary outcomes. Therefore, integration of robust genetic and novel population data, merged with historical information, could significantly aid the trajectory of future Ganges dolphin evolutionary potential. We recommend an integrative approach of demographic, genetic, and environmental aspects, an essential combination to improve our understanding of the future of this charismatic species in the GBMK Basin.

## AUTHORS' CONTRIBUTIONS

Each named author has substantially contributed to conducting the underlying research and drafting this manuscript.

## Data Availability

The authors declare that all data supporting the findings of this study are available within the article (Tables 2, 3, 4).
